# Uncovering emergent phenotypes in endothelial cells by clustering of surrogates of cardiovascular risk factors

**DOI:** 10.1038/s41598-022-05404-7

**Published:** 2022-01-25

**Authors:** Iguaracy Pinheiro-de-Sousa, Miriam H. Fonseca-Alaniz, Samantha K. Teixeira, Mariliza V. Rodrigues, Jose E. Krieger

**Affiliations:** Laboratório de Genética e Cardiologia Molecular, Instituto do Coração (InCor), Hospital das Clínicas, Faculdade de Medicina, Universidade de São Paulo (HCFMUSP), São Paulo, SP Brazil

**Keywords:** Atherosclerosis, Vascular diseases

## Abstract

Endothelial dysfunction (ED) is a hallmark of atherosclerosis and is influenced by well-defined risk factors, including hypoxia, dyslipidemia, inflammation, and oscillatory flow. However, the individual and combined contributions to the molecular underpinnings of ED remain elusive. We used global gene expression in human coronary artery endothelial cells to identify gene pathways and cellular processes in response to chemical hypoxia, oxidized lipids, IL-1β induced inflammation, oscillatory flow, and these combined stimuli. We found that clustering of the surrogate risk factors differed from the sum of the individual insults that gave rise to emergent phenotypes such as cell proliferation. We validated these observations in samples of human coronary artery atherosclerotic plaques analyzed using single-cell RNA sequencing. Our findings suggest a hierarchical interaction between surrogates of CV risk factors and the advent of emergent phenotypes in response to combined stimulation in endothelial cells that may influence ED.

## Introduction

Chronic exposure to well-known cardiovascular (CV) risk factors, including lipids, high blood pressure, tobacco usage, physical inactivity, and diabetes, profoundly affects endothelial function^1^. Surrogates of cardiovascular risk factors are instrumental as biomarkers, drug targets, and tools to investigate their influence on endothelial dysfunction (ED), a critical element for vascular diseases. A large body of evidence suggests that CV risk factors have individual or cumulative effects contributing to the development or worsening of ED, although the complex interplay leading to vascular derangements is poorly understood^2^. Abnormal blood flow, associated with arterial branching and vessel wall irregularities^3,4^, chronic inflammatory states^5,6^, increased oxidized lipids^7,8^ exposure or hypoxia^9,10^ have been shown to affect endothelial and vascular function.

The heterogeneity of endothelial cells (EC) between the vascular bed and tissues must also be considered, especially in light of recent data obtained from systematic global gene expression analyses using single-cell RNA-seq^11,12^. In the murine EC atlas, 78 sub- clusters of ECs were identified based on more than 32,000 ECs from tissues, including brain, testis, liver, spleen, small intestine, colon, skeletal muscle, and heart. These data indicate that the tissue source, as opposed to the type of vascular bed in the blood vessel network, is a critical component that explains most of the heterogeneity of the EC molecular signature^12^. In this context, a significant limitation of current knowledge is the lack of studies using representative ECs from diverse tissues or associated with a particular phenotype. This is highlighted by the frequent use of human umbilical vein endothelial cells (HUVEC) as a model. These cells are easily accessible and facilitate comparing results across studies; however, they are limited to capturing the broad tissue specificities or represent the phenotype of interest. Indeed, HUVEC represents about 44% of the 360 datasets available in The Endothelial Cell Database (EndoDB)^13^ repository (https://endotheliomics.shinyapps.io/endodb/)^13^.

**Figure 1:**
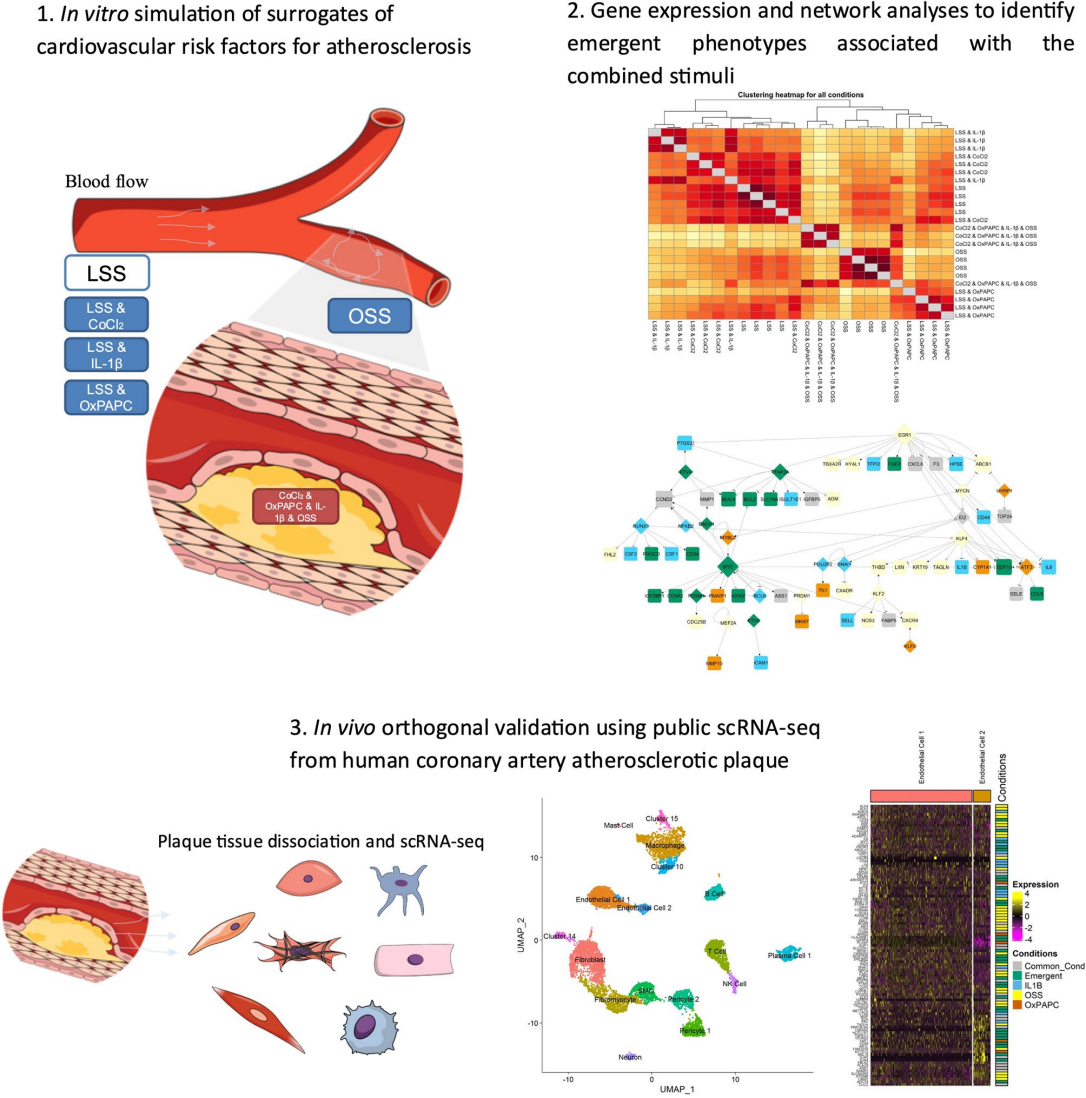
Overall workflow for the identification and validation of emergent phenotypes associated to the combined exposure to surrogates of CV risk factors such as hypoxia (CoCl_2_), Oxidized lipids (OxPAPC), inflammation (IL-1β) and OSS on human coronary artery endothelial cells (HCAEC). 1 Experimental design for in vitro exposure of HCAEC to individual or combined stimuli. 2 Global gene expression and gene regulatory network analyses. 3 Orthogonal validation using scRNA-seq data from human coronary atherosclerotics plaques (dataset GSE131778). The cartoons were created using the Mind the Graph platform (www.mindthegraph.com) and data presented in the manuscript to illustrate the overall workflow.

To address some of these questions, we used human coronary artery endothelial cells (HCAEC) to model the effect of surrogate CV risk factors using global endothelial gene expression. We evaluated the roles of chemical hypoxia (CoCl2), oxidized lipids (OxPAPC), inflammation (IL-1β), and oscillatory shear stress (OSS) in global gene expression using laminar shear stress (LSS) as a reference to establish the hierarchy of cellular response to these stimuli and the impact of the combined effect of all stimuli, which is frequently observed in patients with cardiovascular disease (Fig. 1). Our findings revealed new insights into stimulus-dependent EC gene expression and cellular processes associated with the individual or combined disturbances that may contribute to ED.

## Results

### The effect of surrogate CV risk factors on HCAEC global transcriptomics

We assessed the global transcriptomics in HCAEC exposed individually to CoCl_2_, OxPAPC, IL-1β, and OSS or the combined effect of all stimuli using the cells maintained on LSS as the reference (Supplementary Fig. 1a). We first verified the ability of each stimulus to activate or inhibit downstream associated biomarkers: activation of HIF-1α in response to chemical hypoxia (CoCl2, 150 μM); increased *ATF3* and reduced *CD36* mRNA expression by OxPAPC (50 μg/ml); increased VCAM-1 protein expression by IL-1β (10 ng/ml)-induced inflammation and misalignment of the ECs exposed to OSS (± 5 dyn/cm^2^ at 1 Hz). As shown in Suppl. Figure 1b-e, these surrogates of cardiovascular risk factors altered downstream endothelial biomarkers as expected under the present experimental conditions.

**Figure 2:**
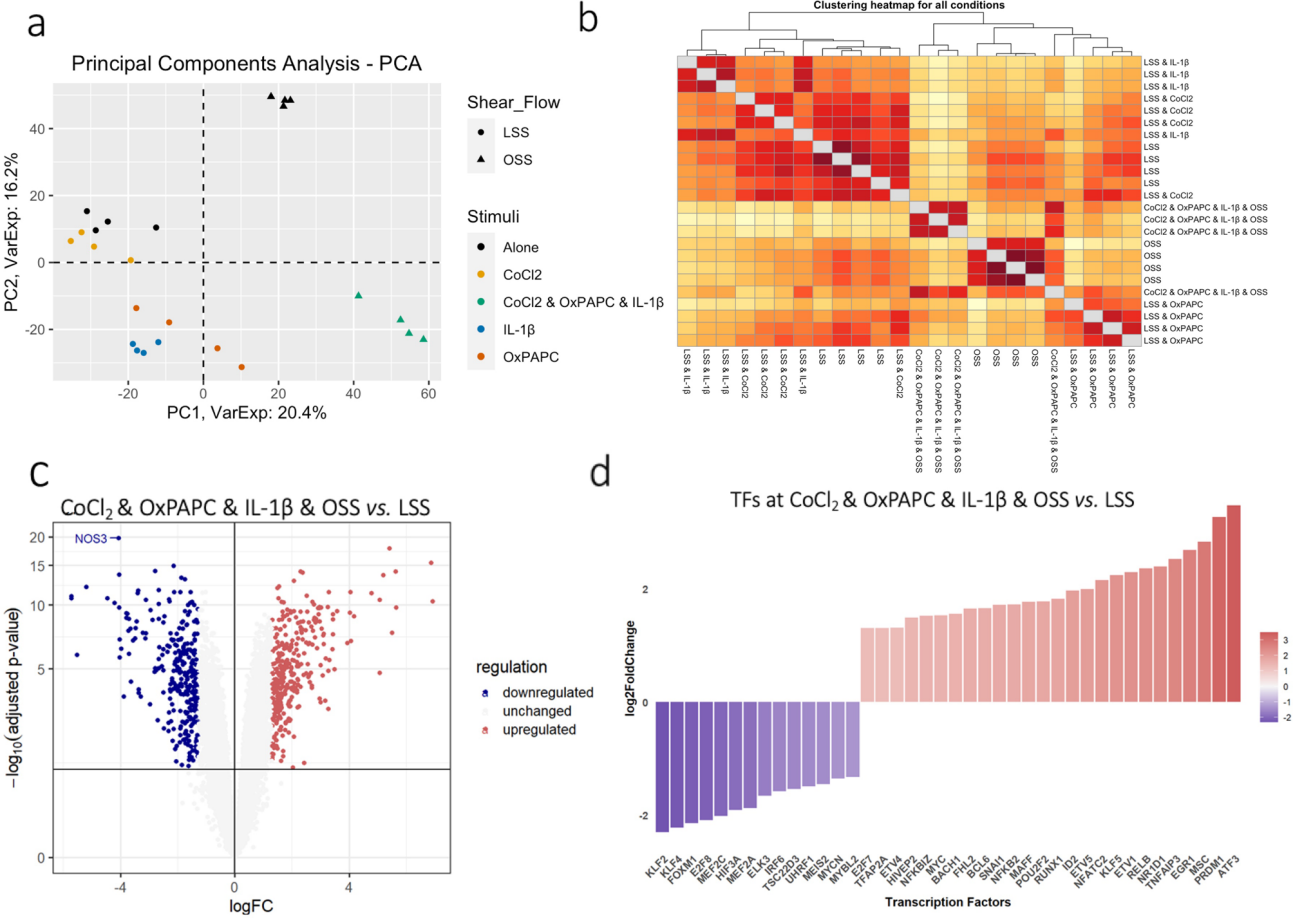
Gene expression profile revealed the hierarchical contribution of the CVD risk factors CoCl_2_, OxPAPC, IL-1β and OSS and key TFs relevant to endothelial function. (**a**) Principal Component Analysis (PCA) of gene expression profile from each sample showed that the PC1 explained 20.4% of the variation while PC2 16.2%. (**b**) Hierarchical clustering heatmap using Euclidean distance as a measured parameter revealed details of hierarchical contribution by each condition according to their distance to the LSS. (**c**) Volcano plot showed the differentially expressed genes (DEGs) when comparing the clustered risk factors vs. LSS. The DEGs were considered as adjusted *p*-value < 0.05 and |Log2foldchange|> 1.3. (**d**) TF identified among the DEGs and their respective log2 foldchange. Genes in blue are downregulated and in red are upregulated.

The effects of each stimulus or the combined exposure on HCAEC global gene expression assessed by principal component analysis (PCA) are shown in Fig. 2a. PC1 and PC2 explain almost 37% of the variance of the expression data in response to all experimental perturbations. The PC1 highlights the critical influence of flow patterns on ECs, dividing the data between samples exposed to either LSS or OSS, while PC2 separates the influence of inflammation, oxidized lipids, and the combined stimuli from all other conditions. Examination of PC1 and PC2 indicates that the combined stimulus perturbation is separated from all individual perturbations, consistent with the appearance of emergent phenotypes not anticipated by the sum of individual stimuli. The HCAEC exposed to LSS clustered with CoCl_2_, whereas the OxPAPC and IL-1β clustered together in a third group, leaving OSS samples isolated as the fourth group (Fig. 2a). The heatmap in Fig. 2b reveals the details of the hierarchical contribution of each condition using the Euclidean distance and the LSS as reference. The two-dimensional graph depicts the samples with the combined stimulus as the most distant (yellow), consistent with the PCA (Fig. 2a). Next, OSS and IL-1β were the individual stimuli more distant from the LSS, followed by OxPAPC and CoCl_2_ stimuli. These findings suggest that the more distant the samples are from the LSS, the more significant each stimulus's impact modulates the global HCAEC transcriptome. This image is consistent with a hierarchical influence of the various stimuli to alter gene expression, causing or altering endothelial dysfunction (ED). CoCl_2_, OxPAPC, IL-1β, OSS, and the combined exposure display a graded impact on changing global gene expression (Fig. 2b).

We identified 620 differentially expressed genes (DEGs) between the two extreme conditions, the combined stimuli (CoCl_2_ & OxPAPC & IL-1β & OSS) versus the LSS control group. There were 297 DEGs that were downregulated and 323 that were upregulated (Fig. 2c), in which the *NOS3* gene*,* a canonical vasodilator associated with the nitric oxide (NO) production^14^, was the most significant downregulated gene. This finding is consistent with the notion that decreased NO production is associated with ED (Fig. 2c). The list of all DEGs is displayed in Supplementary Table 1.

Among the DEGs, we observed 40 differentially expressed transcription factors (TFs) (14 downregulated and 26 upregulated) that may be critical to governing the combined stimulus-induced downstream responses in HCAEC (Fig. 2d). The shear stress-sensitive TFs *KLF2* and *KLF4* were downregulated, while the TFs *NFKBIZ, NFKB2, NFATC2,* and *RELB* and their chemokine targets *CXCL1/3/4/5/6/8* were upregulated (Supplementary Table 1). *MYC*, associated with proliferation, cell growth, and differentiation, was upregulated in the combined stimulus group^15,16^. *EGR1* that is known to mediate downstream hypoxia response, was also upregulated^17^. Although the upregulation of *HIF1A* gene expression was not observed, its protein was increased, as shown in Supplementary Fig. 1b. *ATF3* gene, known to be activated by OxPAPC^18^*,* was upregulated in the combined stimulus group, similar to observed when we exposed static ECs only to OxPAPC (Supplementary Fig. 1c).

### The HCAEC global gene expression response to the combined stimulus is different from the sum of each stimulus

**Figure 3:**
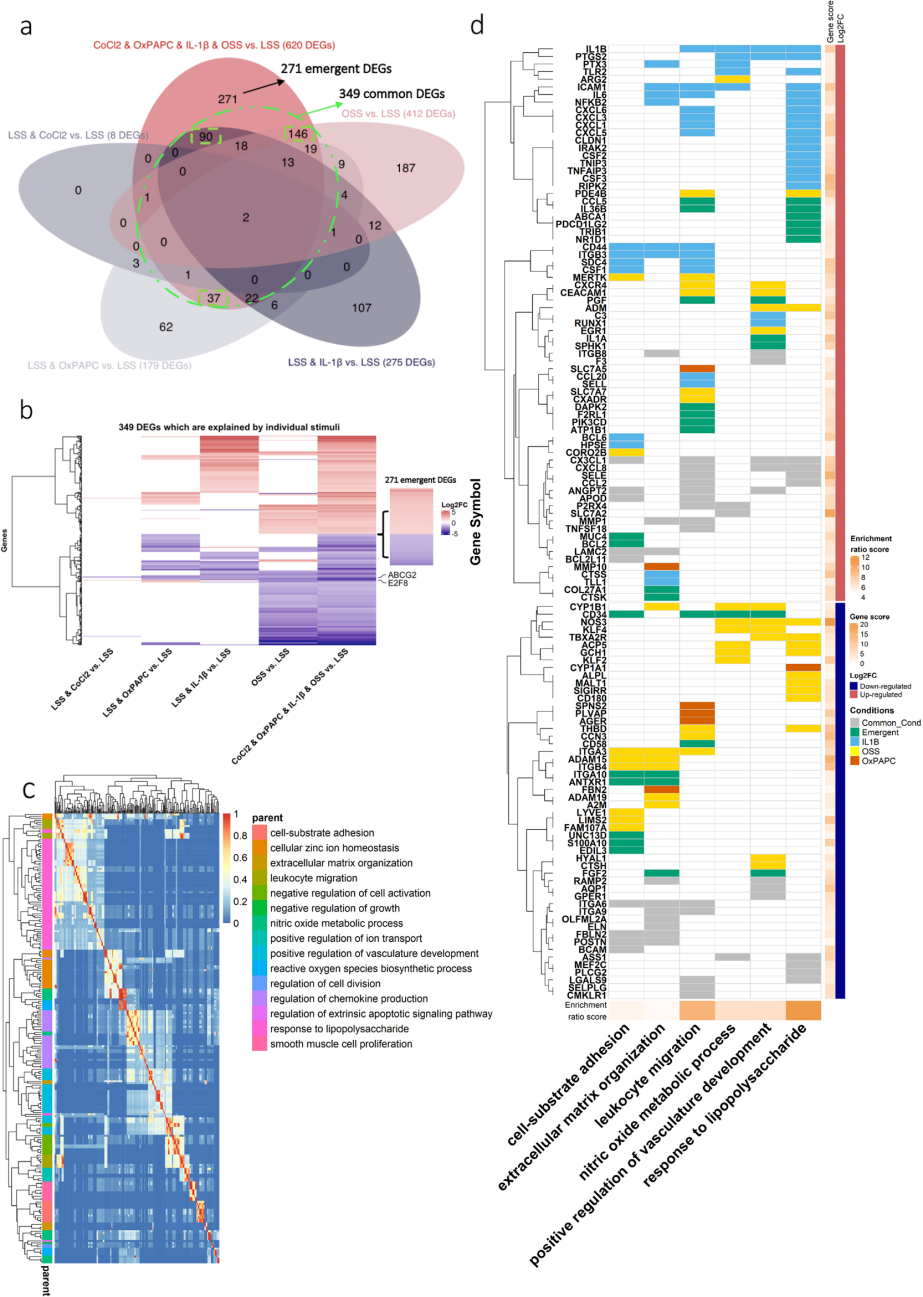
Dysregulated DEGs distribution on individual and clustered risk factors followed by GO map of biological process enriched for the 620 DEGs of the clustered risk factors vs. LSS. (**a**) Venn diagram of DEGs from each condition. The DEGs were considered as adjusted *p*-value ≤ 0.05 and |Log2foldchange|≥ 1.3; black circle and black arrow regard the unique DEGs from the clustered risk condition; dashed green circle and green arrow regard to the commonly shared genes of the clustered risk factors with each stimulus; dashed black line for unique stimuli-dependent genes, 146 DEGs for OSS, 90 for IL-1β and 37 for OxPAPC. (**b**) Heatmap of 349 DEGs shared among the DEGs from each stimulus. (**c**) *Heatmap* of the similarity matrix of enriched GO terms (biological process) for the clustered risk factors DEGs. Redundancy analysis was applied to identify and keep the most representative term from the redundant terms (cutoff = 0.7 and adjusted *p*-value ≤ 0.05). (**d**) Canonical enriched terms in CoCl_2_ & IL-1β & OxPAPC & OSS vs. LSS (121 DEGs) and the source of the stimulus individual (represented by colours light blue, yellow and orange), common (grey), more than one stimulus, and if it is emergent, meaning that the gene is only altered if all four stimuli are combined (green). Gene score and enrichment risk score were calculated by − log_10_(adjusted *p-*value).

Using LSS as a reference, we compared the HCAEC exposed to each stimulus and the combined stimulus for 48 h (Supplementary Fig. 2a-b). Considering the differential expression analysis of individual stimuli, we identified 8, 179, 275, and 412 DEGs associated with CoCl_2_, OxPAPC, IL-1β-induced inflammation, and OSS, respectively (Fig. 3a, Supplementary Fig. 2c-g). Among the 620 DEGs affected by the combined stimulus condition compared to the control LSS, 349 were also affected by one or more individual perturbations (Fig. 3a,b [within the dashed green line]). In contrast, 271 DEGs were exclusively associated with the combined stimulus condition. Among the common 349 DEGs, we observed that OxPAPC uniquely modulated 37 DEGs, 90 were associated with IL-1β-induced-inflammation stimulus, and 146 were modulated only by OSS (Fig. 3a [green squares] and Supplementary Fig. 2c)*.*

The overall influence of each stimulus is depicted in Fig. 3b, indicating that the global gene expression changes individually where the CoCl_2_ stimuli were the smallest. In contrast, OxPAPC and OSS had a greater influence on suppressing gene expression, and IL-1β- induced inflammation primarily increased gene expression. Interestingly, *E2F8* and *ABCG2* were both downregulated, and they were the only DEGs modulated in all conditions examined (Fig. 3b). The TF E2F8 regulates angiogenesis through activation of VEGFA in cooperation with HIF-1α^19^, while *ABCG2* is associated with the cardiac repair of myocardial infarction and ECs survival^20^.

In summary, the individual stimuli did not recapitulate the condition in which all stimuli were combined. Moreover, 44% of DEGs (271) were exclusively associated with the clustered condition, an emerging pattern likely to be lost when each stimulus is assessed individually.

### DEGs and canonical pathways that may contribute to CV risk factors associated with ED

We used Gene Ontology (GO) enrichment analysis to gain insight into biological processes on the 620 DEGs induced by the combined stimulus condition. We grouped the GO terms based on their semantic similarities to minimize the redundancy among the enriched sets. Then, the most representative term was selected according to the highest score within each group of redundant terms. The similarity matrix with the redundant GO terms and their representative term (parent) are represented in Fig. 3c as a heatmap. The findings suggested enriched canonical pathways associated with ED and atherosclerotic plaque formation. The representative groups were as follows: leukocyte migration (green cluster), response to lipopolysaccharide (LPS) and smooth muscle cell proliferation (purple and pink clusters); NO metabolic process, positive regulation of vasculature development, and reactive oxygen species (ROS) biosynthetic process (blue cluster); and cell-substrate adhesion and extracellular matrix (ECM) organization (orange to brown clusters). The GO terms associated with each representative group are displayed in Supplementary Fig. 3a. Figure 3d illustrates six representative processes, showing the DEGs involved and how they are affected by each stimulus or the combined condition. The complete map, including the 15 grouped GO terms, is presented in Supplementary Fig. 3b. It is important to note that the specific DEGs are not always the same even though each stimulus or combined effect influences common processes.

#### Nitric oxide (NO) and ROS

Decreased NO bioavailability and increased ROS production pathways are enriched in our data and are a hallmark of endothelial dysfunction^21,22^ (Fig. 3c,d, and Supplementary Fig. 3a and b). In the NO metabolic process term, the *ARG2* gene, upregulated in the clustered condition, is known for suppressing NO production by competing with the L-arginine substrate^23^. Downstream, L-arginine production might also be impaired by the downregulation of the enzyme responsible for its synthesis (*ASS1*, arginosuccinate synthase)^24^. *NOS3* and its primary activator *KLF2* were both downregulated in the clustered condition in comparison to LSS. *GCH1* associated with positive regulation of NOS3^25^ was also downregulated. CYP1B1 was also downregulated in the clustered condition, and its inhibition was associated with increased oxidative stress and decreased eNOS^26^. *ARG2, NOS3, KLF2,* and *CYP1B1* were only modulated by OSS stimulus (Fig. 3d from top to bottom, in yellow), while *ASS1* (in gray) was suppressed by the stimuli OxPAPC, IL-1β, and OSS. The upregulation of pro-inflammatory genes such as *ICAM1*, *TLR2,* and, especially, *PTGS2,* with its peroxidase activity, was previously associated with induction of oxidative stress^27^ and, consequently, increased ROS production (Fig. 3d top, in light blue). Interestingly, except for the *ASS1* gene, genes regulated by NO production were mainly affected by the OSS stimulus (Fig. 3d bottom, in yellow), while genes responsible for oxidative stress induction were predominantly modulated by IL-1β inflammatory stimulus (Fig. 3d top, in light blue).

#### Inflammatory response and adhesion molecules

ECs respond to LPS via the activation of cytokines, chemokines, and adhesion molecules^28,29^. We observed critical pro-inflammatory gene activation, including *IL6*, *CXCL1/3/5/6, TLR2, NFKB2,* and *RIPK2* (Fig. 3d top, in light blue). Adhesion molecules such as E-selectin (*SELE—*in gray), *ICAM1, CD44, SDC4,* and *CSF1* were upregulated and are associated with leukocyte adhesion and transmigration consistent with the growth plaque phenotype^30^ (Fig. 3d, in light blue). Even though IL-1β induces the majority of the DEGs involved in inflammatory pathways, we also observed a pro-inflammatory role of OSS (*CX3CL1, SELE,* MCP1 (*CCL2*), and OxPAPC (*CXCL8*) (Fig. 3d middle, in gray).

Interestingly, the emergent genes involved in cell-substrate adhesion, leukocyte migration, and response to LPS terms were not directly related to activation of the immune system except by *CCL5* and *IL36B,* both upregulated (Fig. 3d top, in green)*.* The upregulated genes *ABCA1* and *TRIB1* were associated with increased cholesterol uptake^31,32^. PD-L2 (*PDCD1LG2*), upregulated, and *CD58,* downregulated, were associated with suppression of T cell-mediated immune responses^33,34^ and stimulation of T cell, respectively^35,36^ indicating negative feedback on T cell inflammatory response. Finally, we also observed a cell survival phenotype with upregulation of *DAPK2, BCL2*, *MUC4,* which primarily promotes cell growth and apoptosis resistance^37–39^, followed by the upregulation of PI3K (*PIK3CD*), which is associated with cell growth, proliferation, and metabolism^40,41^ (Fig. 3d top to middle, in green). It appears that the emergent DEGs, in addition to their role in inflammatory modulation, are associated with lipid uptake, cell growth, and survival.

#### Vessel remodeling and angiogenesis

The natural progression of atherosclerotic lesions is accompanied by varying degrees of matrix synthesis and breakdown, contributing to vascular remodelling^42,43^. Our data showed that ECM components and matrix-degrading proteases were modulated: elastin (*ELN*), fibulin (*FBLN2*), and fibrillins (*FBN2*) were all downregulated, while laminin (*LAMC2*) and collagen (*COL27A1*) were upregulated; metalloproteinases (*MMP1/10*) were upregulated, whereas disintegrin and metalloproteinase (*ADAM15/19*) were both downregulated. These genes were modulated primarily by more than one condition (Fig. 3d and Supplementary Fig. 3b, in gray) except by *MMP10* and *FBLN2* that were uniquely modulated by OxPAPC and *ADAM15/19* by OSS.

Increased angiogenesis processes^44,45^ follow excessive expansive remodeling, intimal thickening, and ischemia. In this context, we observed activation of angiogenic genes (*PGF, PTGS2, ADM, CEACAM1*, and *ANGPT2)* in the clustered condition compared to LSS (Fig. 3d top to the middle). *PGF* was modulated only by the combined stimuli, whereas *PTGS2* was IL-1β stimulus-dependent. *ADM* and *CEACAM1* were regulated by OSS, whereas *IL-1β* and *OxPAPC* modulated *ANGPT2*. Interestingly, the activation of the vasculature development pathways occurred mainly by genes modulated by OSS (*CXCR4* and *EGR1* were upregulated, and *NOS3*, *KLF2,* and *KLF4* were downregulated) (Fig. 3d top and bottom, in yellow). Indeed, it has been demonstrated that OSS stimulates angiogenesis while LSS stabilizes vessel remodeling by inhibiting angiogenesis sprouting^46,47^. Our data suggest that OSS plays a critical role in regulating vasculature development rather than any other stimulus alone or in combination.

#### Coagulation and thrombogenesis

The shift from low-risk to high-risk plaques usually accompanies ECM degradation, plaque erosion, or rupture with thrombosis of the plaque surface^48–50^. Three critical genes associated with coagulation cascade were activated and depicted in the positive regulation of vasculature development and cell- substrate adhesion terms (Fig. 3d). Tissue factor (*F3* in gray), upregulated by OxPAPC and IL-1β, initiates blood coagulation by forming a complex with circulating factor VII or VIIa^51^; heparanase (*HPSE* in blue), upregulated by IL-1β, is a pro-coagulant that increases the generation of activation factor X in the presence of tissue factor and activation factor VII^52^, and *F2RL1,* an emergent gene in green, is involved in the coagulation of factor X^53^. Anti-thrombogenic genes were downregulated, including thrombomodulin (*THBD*) and plasminogen activator (*PLAT*), both modulated by OSS (Fig. 3d and Supplementary Fig. 2c). Our data suggest that pro-coagulant factors were activated primarily by inflammatory IL-1β stimulus while OSS suppresses the anti- coagulant genes.

Overall, these data demonstrate that global gene expression response to key surrogates of CV risk factors recapitulates known ED biological processes. Moreover, we provide a yet undescribed context-dependent view regarding the role of individual stimuli and an emergent pattern that arises from the combined stimulus condition, which is usually present in cardiovascular patients.

### Transcriptional regulatory ED network

**Figure 4:**
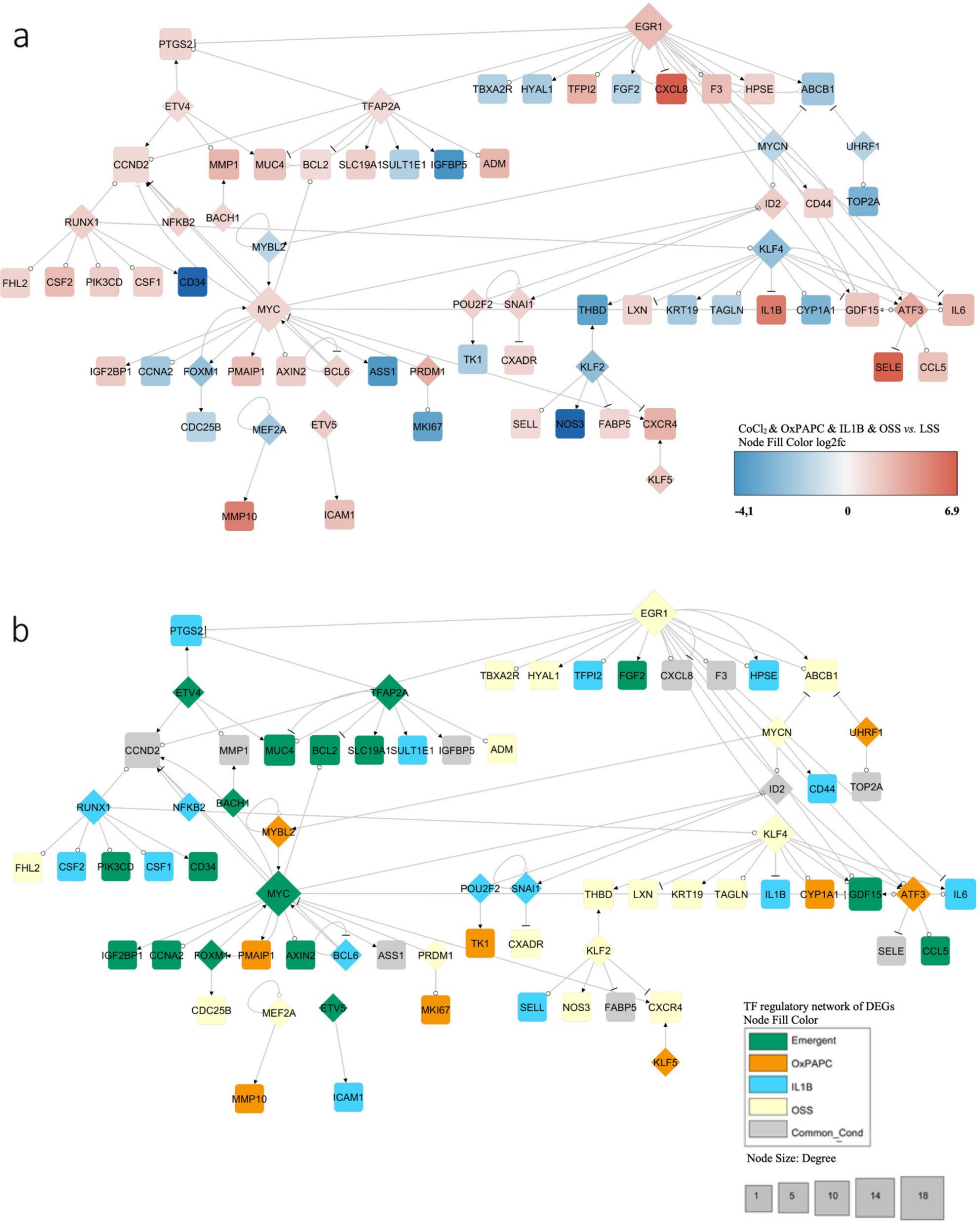
TF-DEGs regulatory network of the combined stimuli condition. (**a**) Using the TRRUST database (version 2), the transcription factors (TFs) were mapped to their published transcriptional targets. The identified transcriptional network was further filtered based on whether the TF and their targets were a DEG in CoCl_2_ & OxPAPC & IL-1β & OSS vs. LSS (adjusted *p*-value ≤ 0.05 and |Log2foldchange|≥ 1.3). The diamond shape is TF and the square is the DEG target. The edges mean arrow (activation), circle (unknow) and bar (repression). Node size according to the degree. Genes in blue are downregulated, genes in red upregulated. (**b**) TF-DEGs regulatory network and the condition which they also are differentially expressed. Green is a modulated emergent gene; Orange is an OxPAPC modulated gene; Light blue is an IL-1β modulated gene; Yellow is an OSS modulated gene; Gray is when the gene is modulated in more than one condition.

To understand the functional relationship between the combined versus the individual surrogates of CV risk factors in HCAEC response, we generated a gene regulatory network to map the TFs to their target DEGs using the TRRUST database^54^. We first used the 620 DEGs from the combined condition, in which the TF-DEG network was composed of 71 DEGs (22 TFs among them) with 94 interactions (Fig. 4a). The three highest connected nodes were *EGR1*, *MYC,* and *KLF4,* with 18, 17, and 11 pairs of interactions, respectively. In Fig. 4b, we highlight the role of each condition on these responses: OSS (yellow), the emergent effect from the combined stimulus condition (green), and IL-1β-induced inflammation (blue) were critical factors modulating 19, 17, and 16 genes, respectively, of the generated gene regulatory network. The TF-DEG regulatory networks of the individual stimuli are displayed in Supplementary Fig. 4.

#### EGR1, KLF4, and KLF2 are key TFs nodes modulated by OSS

Among the individual stimuli, OSS had a significant effect on ED, and it appears that the TFs that play a Critical role are *EGR1* (upregulated) and *KLF2,* and *KLF4* (downregulated) (Fig. 4a,b, in yellow). *EGR1* targets include the upregulated genes *PTGS2, CCND2, TFPI2, CXCL8, F3, HPSE, GDF15, ATF3,* and *IL6*, and the downregulated genes were *TBXA2R, HYAL1, FGF2,* and *ABCB1* (Fig. 4a). Nevertheless, only *CCND2, TBXA2R, HYAL1,* and *ABCB1* were differentially expressed by the OSS condition (Fig. 4b and Supplement Fig. 4d). This analysis inferred the genes regulated by a given TF and confirmed whether its targets were differentially expressed by the stimuli modulating the TF (Fig. 4b).

*EGR1* was the most critical hub in the network. This TF has been recently identified as the most enriched in regulatory regions in human vein and artery endothelial cells subjected to OSS^55^ and has been predicted to act as a significant regulator of ECs under OSS^56^. *KLF2* and *KLF4* are significant regulators of anti-inflammatory response and maintenance of vascular integrity^57^. Their repression may increase leakage, formation of blood clots, and altered vascular tone^57^. Inflammatory *KLF4* targets were all upregulated: *LXN*, *IL1B,* and *IL6* (Fig. 4a). *LXN* was the only one modulated by OSS. *KLF2* pro-atherogenic target *CXCR4* was upregulated while the anti-atherogenic target *NOS3* was downregulated, and they were all OSS stimulus-dependent (Fig. 4a,b). KLF2 and KLF4 regulated the anti-thrombogenic gene THBD (Fig. 4a).

Our data are consistent with a large body of evidence indicating that *EGR1*, *KLF4,* and *KLF2* are master regulators of ECs under shear stress^55,57,58^. These TFs increase *NOS3* expression, reduce oxidative stress, and inhibit chemokines receptors like *CXCR4*. A more detailed and similar network with all OSS stimulus-dependent DEGs is shown in Supplementary Fig. 4d.

#### Proliferative emergent behavior and its impact on inflammatory stimuli

The combined stimulation condition uncovered the MYC node. This response suggests that clustering of risk factors elicits emergent properties that the individual perturbations cannot recapitulate. *MYC* targets comprise *CCND2*, *CCNA2*, *FOXM1,* and *BCL2* associated with cell cycle and apoptosis resistance^16,38^, where *CCND2* and *BCL2* are upregulated and *CCNA2* and *FOXM1* are downregulated (Fig. 4a). The emergent hubs include *MYC* and *TFAP2A,* shown in green in Fig. 3b. The IL-1β-stimulated TFs *RUNX1* and *BCL6* promote angiogenesis^59,60^ (Fig. 4a,b). The *RUNX1* targets *CSF1/2* and *PIK3CD* are involved in cell survival, growth, proliferation, and inflammatory responses. *CSF1/2* were activated by IL-1β stimulus, while *PIK3CD* (upregulated) is an emergent gene and well-known activator of the mTOR pathway signalling^61,62^ (Fig. 4a,b).

Curiously, when we performed the TF-DEGs gene regulatory network for each stimulus, the critical network was driven by IL-1β-inflammation stimulus, where the *NFKB1* was the central hub (Supplementary Fig. 4c). Nevertheless, *NFKB1* was not differentially expressed in the clustered condition, nor were many of its targets (*BMP2*, *CXCL2*, *LCN2*, *VCAM1,* and *IRF1*). Furthermore, most of the *NFKB1* gene targets had their log2 fold-change decreased in the clustered condition compared to the IL-1β- inflammation stimulus alone (Supplementary Fig. 4e). It appears that the clustered condition suppresses part of the inflammatory response, and it gives space to a proliferative behavior and EC survival phenotype.

### Validation of the HCAEC data using single-cell RNA-seq from human coronary atherosclerotic plaque

**Figure 5:**
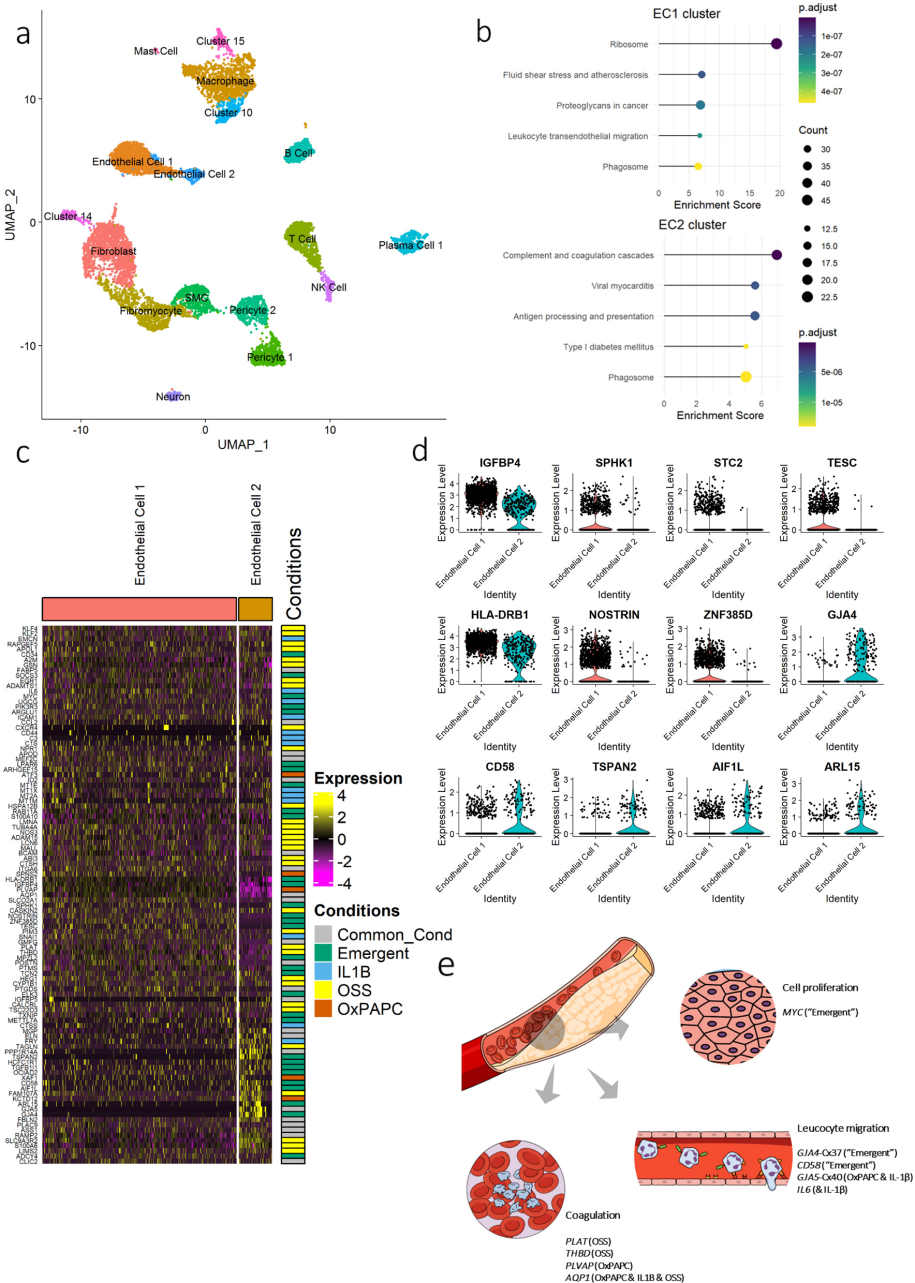
scRNA-seq of human coronary artery atherosclerotic plaque identified two endothelial cells (EC) clusters which are defined by the clustered risk factors DEGs. (**a**) UMAP visualization of clustering identified 17 cell populations (n = 10,671 cells). (**b**) KEGG enrichment analysis of the two EC clusters using the cluster marker differentially expressed genes between a cluster and all remaining cells. DEG were considered for adjusted *p*-value ≤ 0.05 and |Log2foldchange|≥ 0.25. (**c**) Heatmap of the 103 clustered risk factors DEGs expression levels from each endothelial cell of the two clusters followed by the condition in which they are differentially expressed. (**d**) Violin plot of emergent DEGs which are differentially expressed in EC2 vs. EC1. (**e**) Schematic illustrating the key dysregulated pathway in the clustered risk factors which are recapitulated in human atherosclerotic plaque. (**e**) Created in the Mind the Graph platform (www.mindthegraph.com).

Our in vitro data indicated that the combined stimulus condition is critical to uncover emergent phenotypes that may disrupt the endothelial cell compensatory mechanisms leading to disease states. We then determined whether this was the case in an in vivo chronic and inflammatory atherosclerotic plaque condition. We re-analyzed a public single-cell RNA-seq dataset obtained from human coronary artery atherosclerotic lesions from patients with clustered risk factors (GSE131778)^63^. We analyzed 10,671 cells and identified 17 cell clusters (Fig. 5a and Supplementary Fig. 5a). We used *PECAM1* (CD31) expression to identify the ECs clusters (Supplementary Fig. 5b and Supplementary Table 2). The cell type clusters were annotated by the top defining genes in each cluster (Supplementary Fig. 5c).

We first performed a pathway enrichment analysis using the DEGs between each cluster against all the remaining ones. The DEGs (843) observed in the EC1 cluster were mainly involved in ribosome-associated pathways, fluid shear stress in atherosclerosis, cancer proteoglycan, and leukocyte transendothelial migration. In contrast, the DEGs (644) in the EC2 cluster were related to coagulation cascade, viral myocarditis, and type I diabetes mellitus (Fig. 5b and Supplementary Table 2).

Our primary goal was to determine whether the DEGs associated with the combined stimuli were involved in the transcriptomic profile associated with the two EC clusters (843 and 644 DEGs for clusters EC1 and EC2, respectively) and uncover the potential role of the emergent genes in these processes. First, 17% of the DEGs previously identified (103/620 DEGs of the combined stimulus condition) were among the DEGs defining these two EC clusters. Figure 5c shows how these 103 DEGs were influenced by each stimulus in both EC clusters based on our in vitro assays. The OSS stimulus modulated 32 genes, 31 genes displayed the emergent pattern, followed by 22 differentially expressed genes in more than one condition, and 13 and 5 DEGs were modulated only by IL-1β and OxPAPC, respectively (Fig. 5c). Interestingly, it appears that these validated 103 DEGs are critical genes defining the EC1 and EC2 clusters in the heatmap (Fig. 5c middle to bottom). For example, *IGFBP4*, *PLVAP,* and *AQP1* are less expressed in EC2 than EC1, while *ARL15*, *GJA5,* and *GJA4* are more expressed in EC2 than EC1 (Fig. 5c).

Next, we identified 673 DEGs between EC cluster 2 vs. 1 (Supplementary Fig. 5d and Supplementary Table 3). Examining them against the 620 DEGs from the in vitro combined stimuli condition, we identified 71, with 23 displaying the emergent pattern. To highlight these 23 emergent DEGs in human coronary artery plaque, we plotted them in each EC cluster (Fig. 5d and the complete list in Supplementary Fig. 5e). *IGFBP4, SPHK1, STC2, TESC, HLA-DRB1,* and *NOSTRIN* were downregulated in EC2 compared to EC1. These genes are involved with a broad array of cellular functions. *IGFBP4* and *STC2*, for example, have been associated with cell growth and glucose uptake^64^ and angiogenesis induction^65^, respectively. *SPHK1* was associated with endothelial permeability^66^, while *TESC* was associated with cardiomyocyte hypertrophy^67^. *HLA-DRB1* was associated with ED in patients with rheumatoid arthritis^68^, while *NOSTRIN* was associated with decreased eNOS activity^69^ (Fig. 5d). *MYC* was upregulated at EC1 only compared to all other clusters (Fig. 5c and Supplementary Fig. 5f.).

In contrast, we observed that *GJA4, CD58, TSPAN2, AIF1L,* and *ARL15* were upregulated in EC2 compared to EC1. *GJA4* and *CD58* have been associated with increased cell–cell junction and leukocyte migration^70,71^. *ARL15* overexpression showed a protective effect on the endothelium by increasing NO and decreasing ROS^72^ production. *TSPAN2* appears to play a role in the migration of ECs and SMCs^73^ and has been strongly associated with atherosclerosis in large arteries^74^. However, the role of *TSPAN2* remains to be elucidated^73^. *AIF1L* was associated with increased necrotic core and is a marker of plaque vulnerability^75^.

Understanding the molecular architecture underlying the EC clusters' biological function improves upon evaluating the behavior of the genes in response to the individual stimuli in vitro. *PLAT* and *THBD* (OSS) and *AQP1* (OxPAPC & IL-1β & OSS) were downregulated in EC2 compared to the EC1 cluster (Supplementary Fig. 5f.), suggesting that these cells are more susceptible to thrombosis, consistent with data shown in Fig. 5b. The decreased expression of genes associated with cell–cell junction in EC1, such as *GJA4* (emergent) and *GJA5* (OxPAPC & IL-1β), are consistent with increased adhesion and migration of leukocytes pathway, enriched in the EC1 cluster (Fig. 5b).

These findings suggest that HCAECs under the combined surrogates of risk factors recapitulated, at least in part, the changes in canonical pathways observed in vivo in human coronary atherosclerotic plaque samples analyzed by single-cell RNA-seq. The 103 DEGs from the in vitro experiment overlapped with the DEGs from human atherosclerotic plaque ECs, allowing us to infer the pathways that are preferentially influenced by each stimulus or by the combined stimulation in vitro, further supporting our model. The composition of ECs on the plaque suggests that one cluster displays an inflamed and proliferative susceptible phenotype with increased leukocyte migration (EC1), whereas the cells are more prone to thrombosis EC2 cluster (Fig. 5e).

## Discussion

We showed that DEGs observed in ECs from human coronary artery plaques can be modeled in vitro by exposing HCAEC to surrogates of the main CV risk factors applied individually or in combination. Using this approach, we provide new insights about the ED molecular architecture and the gene networks and inferred cellular processes modified by individual surrogates of CV risk factors, which display a hierarchical influence pattern. Furthermore, we demonstrated for the first time that the combined exposure to surrogates of CV risk factors, as seen in cardiovascular patients, give rise to emergent properties with unique modulation of gene networks and processes, such as the activation of cellular proliferative pathways, that is not recapitulated by the sum of influences from the individual perturbations.

It is well-known that hypoxia^44,76^, OxPAPC^77,78^, IL-1β^1,6^ and OSS^79,80^ are major players in endothelial function/dysfunction. Hypoxia and HIF signaling stimulate EC survival and growth, cell invasion, and glucose metabolism, contributing, in general, to angiogenesis^81,82^. Chemical hypoxia also increases HIF-1α^83^ by preventing its degradation, as we showed in Supplementary Fig. 1b, even though we did not observe differences in HIF-1α expression under this stimulus. It is also known that OSS alters EC metabolism via activating HIF-1α, which induces cell proliferation and inflammation by activating glycolytic enzymes^84^. Interestingly, CoCl_2_ or OSS alone did not achieve this proliferative and angiogenic phenotype, only observed when the stimuli were combined. This response may be explained by the EC compensatory capacity's cumulative perturbations and exhaustion, giving rise to altered phenotypes.

The modification of an established response to individual perturbations or the appearance of an emergent phenotype associated with the combined stimulation were also detected in other canonical responses. We recapitulated the activation of critical inflammatory markers including NF-κβ (*NFKB1*), *ICAM1*, E-selectin (*SELE*), and IL-8 (*CXCL8*) that was observed in LSS cultured HUVEC exposed to IL-1β and TNF-α ^85^. However, when the IL-1β was combined with other stimuli, *NFKB1* no longer changed its expression (Supplementary Fig. 4e), and neither did several of its pro-inflammatory targets (*BMP2, CXCL2, LCN2, VCAM1*, and *IRF1*). Interestingly, this partial suppression of inflammatory response was accompanied by the emergence of a proliferative and cell survival program activated by the proto-oncogenes *MYC* and *BCL2,* with the former being an emergent hub on the TF-DEGs network (Fig. 4b). *MYC* is an exciting target because it has been associated with atherosclerosis by promoting smooth muscle cell proliferation^86,87^, and we showed that it is also upregulated in ECs from human coronary atherosclerotic plaques. We were only able to model this phenomenon because we analyzed the individual and the combined contribution of the stimuli, a likely in vivo scenario where multiple CV risk factors coexist.

Alsaigh et al.^88^ characterized ECs from human carotid artery atherosclerotic plaques using scRNA-seq similarly to the analysis we performed in the endothelial cells from coronary plaques data from a previously reported study^63^. They also identified two EC clusters, one localized on the atherosclerotic core (AC) displaying comparable gene expression changes that we observed in the EC2 cluster (*ACKR1, IL6, IGFBP4, HLA- DRA, HLA-DRB1,* and *NOSTRIN* were downregulated while *MPZL2*, *GJA5, ELN, END1, RHOB* were upregulated) (Supplementary Fig. 5d-f). In contrast, the EC cluster from the adjacent region to the carotid plaque called the proximal adjacent (PA) region, behaved as the EC1 cluster described here. They found that the DEGs that define the AC cluster were enriched in epithelial-mesenchymal transition, angiogenesis, coagulation cascade, and hypoxia pathways, whereas the inflammatory response, MYC targets, and E2F targets pathways defined the PA cluster^88^. Altogether, the results from datasets of scRNA-seq from atherosclerotic plaques in different vascular beds suggest different transition states of ED throughout atherosclerotic lesions. These observations are consistent with our findings, indicating a hierarchy among the effects of different stimuli and the appearance of emergent phenotypes when the stimuli are combined. Thus, varying with the presence and the intensity of the different pathologic stimuli, as seen in different patients, may give rise to several subsets of phenotypes.

Using the global gene expression patterns from in vitro perturbations of ECs and data from human coronary artery plaques, we identified the emergent properties that can arise from multiple non-linear interactions and feedback loops governing EC function/dysfunction. The reductionist in vitro approach was informative for identifying the hierarchy of the various stimuli in altered global gene expression and gene networks, enabling inferences regarding ED processes, including leukocyte adhesion, thrombogenesis, and cell proliferation. Notably, the genes associated with the latter were only identified by the combination of surrogates of CV risk factors, highlighting the difficulties in recapitulating components of complex phenotypes in vitro. The effects of CV risk factor clustering surrogates on ECs are different from the sum of the individual responses, and approximately 44% of DEGs and associated gene pathways are unique to the clustering condition.

Our results suggest that changes in gene networks and inferred cellular processes observed in coronary artery atherosclerotic plaques could be modeled and further understood using in vitro approaches where individual. The combined contribution of different factors can be carefully managed dissected to uncover hierarchic patterns and emergent properties*.* These emergent properties may have significant consequences for fundamental investigations of endothelial (dys)function associated with multiple interactions. One may speculate that, regardless of these characteristics, testing additional samples from different beds and conditions may enable prediction and identification of the main hubs or gene networks associated with specific or combined stimuli enabling the design of targeted therapeutic approaches to prevent or retard plaque development. In summary, we provided evidence of a hierarchical effect between surrogates of CV risk factors and the advent of emergent phenotypes in response to combined stimulation in ECs that may influence ED.

## Methods

### Cell culture and stimulations

HCAEC were acquired from LONZA. The cells were grown in EBM-2MV medium (LONZA) and incubated at 37 °C, 5% CO2. All experiments were carried out between passages 4 and 6. HCAEC were previously treated with cobalt chloride (CoCl2, Sigma-Aldrich), OxPAPC (1-palmitoyl-2- arachidonoyl-sn-glycero-3-phosphorylcholine) (SINAPSE), interleukin 1β (IL-1β) (Thermo Fisher Scientific), laminar shear stress (LSS), or oscillatory shear (OSS) to confirm each stimulus individually. As soon as the cells reached 100% confluence (static condition except by shear stress), they were subsequently individually treated with CoCl2 (150 μM), OxPAPC (curve concentration of 25, 50, and 100 μg/ml) or IL-1β (10 ng/ml) and cultured for 48 h. To confirm the effect of each stimulus, we used the protein expression of VCAM for the IL-1β stimulus, *CD36*, and *ATF3* gene expression for treatment with OxPAPC, and protein expression for HIF1-α to confirm chemical hypoxia induced by CoCl_2_.

For experiments with shear stress, we used the IBIDI equipment (IBIDI). Using the Pump Control software, this equipment simulates, controls, and quantifies the shear force applied to ECs throughout the experiment. The ECs were grown on μ-Slides I Luer 0.4 (IBIDI) coated with 0.1% gelatin. HCAECs were grown to 100% confluence (approximately 100,000 cells/cm^2^) on the slides and incubated overnight under standard static cell culture conditions. The ECs were exposed to a unidirectional atheroprotective shear flow with 20 dynes/cm^2^ (LSS) and an atherogenic, bidirectional at 1 Hz (0 ~  ± 5 dynes/cm^2^) shear flow (OSS) for 48 h. We used the cell alignment as a positive effect of the shear forces.

Once all the stimuli were confirmed, we further experimented with the individual or combined factors as follows: i) cultivation of ECs in μ-Slides I Luer 0.4 overnight; the next day, the cells were coupled to the perfusion set containing 12 ml of EBM-2MV medium; ii) addition of stimuli: IL-1β (10 ng/ml), OxPAPC (50 μg/ml), or CoCl2 (150 μM) individually or combined to the perfusion set and integrated into the pump; iii) the ECs were subjected to either the LSS or OSS for 48 h (Supplementary Fig. 1a). The total RNA was collected after each experiment.

### Reverse-transcriptase quantitative polymerase chain reaction (RT-qPCR)

The ECs were lysed with 1 mL of TRIzol Reagent (Invitrogen). Total RNA after extraction and purification was reverse transcribed using SuperScript IV Reverse Transcriptase (Thermo Fisher Scientific). Real-time PCR was performed using SYBR-Green (Roche) with the QuantiStudio 12 K Flex system (Applied Biosystems). We used GAPDH Ct values to normalize ΔCt. The variation in gene expression between samples was calculated using the ΔΔCt method. The primers are as follows:

- *ATF3 Fow./Rev.:* GCAAAGTGCCGAAACAAGAAG/GCTTCTCCGACTCTTTCTGC
- *CD36 Fow./Rev.:* AGATGCAGCCTCATTTCCAC/AGATGCAGCCTCATTTCCAC

#### Protein extraction and western blotting

The cells were lysed in RIPA buffer (150 mM NaCl, 0.5% sodium deoxycholate, 1% Triton X-100, 0.1% SDS, 50 mM Tris–HCl, pH 8) containing protease and phosphatase inhibitors (Sigma Aldrich), and proteins were quantified using a BCA protein assay kit (Pierce Biotechnology). Equivalent amounts of protein were solubilized in sample buffer (0.5% SDS, 10% glycerol, 0.05% bromophenol blue, 50 mM dithiothreitol, 50 mM Tris, pH 6.8) and subjected to polyacrylamide gel electrophoresis in the presence of sodium dodecyl sulfate using polyacrylamide gels of different concentrations. After electrophoresis, proteins were transferred to polyvinylidene fluoride membranes (PVDF 0.45, Amersham Hybond P). Membranes containing the transferred proteins were incubated first in blocking solution (5% bovine serum albumin and 0.1% Tween 20 in TBS, pH 7.4) for 1 h at room temperature, followed by incubation for 16–18 h in specific primary antibody diluted in blocking solution at 4 °C. The following primary antibodies were used: anti-HIF-1α (1:1000, #3619 Cell Signaling Technology) and anti-VCAM-1 (1:1000, #TA50391, Origene). The membranes were also incubated with primary antibodies specific for GAPDH (1:2000, #ab22555 Abcam) or beta-actin (1:2000, #ab8227 Abcam) used as internal controls (housekeeping). After incubation for 1 h at room temperature with secondary antibody conjugated to horseradish peroxidase, the bound primary antibody was detected using a chemiluminescent image analyzer (ImageQuant LAS 4000 mini, GE HealthCare), and the images were quantified by densitometry using ImageJ software (Wayne Rasband).

#### Microarray gene expression profiling

We used the RNeasy Micro Kit (Qiagen) for total RNA isolation to increase the efficiency of RNA extraction from samples with little material, such as those grown in IBIDI. We follow the manufacturer's protocol (RNeasy Micro Handbook). To generate global gene expression (> 20,000 genes), we used the Human Clariom S Assays (Thermo Fisher Scientific) that detect only the exons present in all expressed isoforms of transcripts known as exons constituting locus of a single gene. The samples were processed in a plate matrix in the GeneTitan TM Microarray System (ThermoFisherScientific). The entire protocol for processing and running the samples followed the standard protocol (GeneChip TM WT PLUS Reagent Kit Manual Target Preparation for GeneChip TM Whole Transcript (WT) Expression Arrays).

#### Microarray analysis and quality control

Using the 24 CEL files generated from the GeneTitan TM Microarray System, the quality control of each file was checked before further analysis. The package used was the array QualityMetrics^89^ to identify abnormal signal intensities (saturation or noise), an average of the variance of the intensity of this signal, and possible outliers. To calculate abnormal intensities, we used the Kolmogorov–Smirnov test between the distribution of the intensity matrix of each array (sample) and the distribution of the grouped data. Another analysis was the standard deviation of the intensities in the matrixes after normalization and transformation to a logarithm scale to identify possible signal saturation. Finally, Hoeffding's D statistic was performed in the joint distribution of M and A for each matrix to identify outliers. M and A were defined as follows: M = log2 (I1) − log2 (I2) A = 1/2 (log2 (I1) + log2 (I2)), where I1 is the intensity of the studied matrix and I2 is the intensity of a "pseudo" matrix that consists of the median between the matrixes. D > 0.15 was considered a threshold to remove outliers. All samples passed the quality control check.

#### Differential expression analysis

The analysis of differential gene expression was performed with the limma package^90^. The analysis was performed using the logarithmic matrix of the signal strength adjusted in a linear model, using the lmFit () function. We apply Bayes empirical statistics to this linear model, calculating a moderate t-test for each gene, calculating the t-value and its corresponding *p*-value for each gene. Concomitantly, an adjustment is made by multiple tests. In this case, the adjustment was made by BH, which is the method of Benjamini and Hochberg to control the false discovery rate. We consider DEGs to be those with an adjusted *p*-value < 0.05 and the absolute value of |Log2Foldchange|> 1.3. The analysis of the transcriptome data was performed using the R platform to analyze and visualize the molecular profile of gene expression, differential expression analysis, pathway enrichment analysis, and GO for biological processes.

#### GO-term enrichment and Kyoto Encyclopedia of Genes and Genomes (KEGG) pathway enrichment analysis

We performed an over-representation analysis to determine whether known biological processes or gene pathways are enriched using a list of DEGs. GO terms and KEGG pathway enrichment analysis was performed using the clusterProfiler^91^ Bioconductor package. Redundancy analysis was applied to identify and keep the most representative GO terms from the redundant terms (cutoff = 0.9 and adjusted *p*-value ≤ 0.05). We calculate a score based on the − log10 (adjusted *p*- value) of the significantly enriched pathways and biological processes. Distances between points represent the similarity between terms. GO terms and KEGG enriched pathways were considered with an adjusted *p*-value less than or equal to 0.05.

#### Transcriptional gene regulatory network

Using the TRRUST database (version 2), the TFs were mapped to their published transcriptional targets. The identified transcriptional network was further filtered based on whether the TF and targets were a DEG (adjusted *p*-value ≤ 0.05 and |Log2Foldchange|≥ 1.3). Node size varied according to the degree. Cytoscape (version 3.7.2) was used to topologically visualize the TF- DEGs regulatory network and Network Analyzer tool to identify the hubs based on high degree and high closeness centrality values.

#### Single-cell RNA-seq public data analysis

The public data (GSE131778) for the scRNA-seq of human coronary artery plaques^63^ are available at the Gene Expression Omnibus database. We used the cell gene expression matrixes to further analysis using the R package Seurat version 3.2.2^92^. We trimmed the cells expressing fewer than 200 genes and genes expressed in fewer than three cells. We filtered out cells that had unique gene counts (nFeature_RNA) over 2,500 or less than 200. Cells containing > 5% mitochondrial genes were presumed to be of poor quality and were also discarded. Cell counts were log-normalized by a size factor of 10,000 RNA counts and then scaled. Next, PCA was applied to the scaled data for dimensionality reduction. We used the FindNeighbors() and FindClusters() functions on the Euclidean distance PCA space to refine the edge weights between any two cells to iteratively group cells with a similar expression pattern. We used UMAP to visualize the identified clusters of cells. We applied the FindMarkers () function to identify the markers that defined each cluster, which automatically, via differential expression analysis, compared a cluster against all others. We kept as gene markers *p*-value ≤ 0.05 and average |Log2Foldchange|≥ 0.25. The top ten cell markers were used to manually annotated each cell type.

#### Statistical analysis

For all statistical analyses, R software (version 4.1.0) was used. For qPCR and western blot, we used Student’s t-test to compare two groups and one-way analysis of variance for multiple comparisons. Statistical significance was considered at *p* < 0.05.

## Supplementary Information

Supplementary Information 1.

Supplementary Information 2.

Supplementary Information 3.

Supplementary Legends.

Supplementary Information 5.

## Data Availability

The raw data (CEL files) of the microarray expression data were deposited at the GEO database with the reference (GSE181822).

## Abbreviations

ATF3: Activating transcription factor 3
CCL2: C–C motif chemokine 2
CV: Cardiovascular
CXCL1: C-X-C motif chemokine 1
CXCL8: C-X-C motif chemokine 8
CXCR4: C-X-C chemokine receptor type 4
CXCR7: C-X-C chemokine receptor type 7
CXCL12: C-X-C motif chemokine 12
DEGs: Differentially expressed genes
ECs: Endothelial cells
ECM: Extracellular matrix
ED: Endothelial dysfunction
FGF2: Fibroblast growth factor 2
GJA4: Gap junction alpha-4 protein
GJA5: Gap junction alpha-5 protein
GO: Gene ontoloty
HCAEC: Human coronary artery endothelial cell
HIF-1α: Hypoxia-inducible factor 1-alpha
ICAM1: Intercellular adhesion molecule 1
IL-1β: Interleukin 1 beta
KEGG: Kyoto encyclopedia of genes and genomes pathway
KLF2: Kruppel-like Factor 2
KLF4: Kruppel-like Factor 4
LSS: Laminar shear stress
MMP1: Matrix metalloproteinase-1
MMP9: Matrix metalloproteinase-9
MCP-1: Monocyte chemoat- tractant protein-1
NOS3: Nitric oxide synthase 3
OSS: Oscillatory shear stress
OxPAPC: Oxidezed 1-palmitoyl-2-arachidonyl-sn-glycero-3-phospholcholine
PECAM1: Platelet endothelial cell adhesion molecule-1
PI3K: Phosphatidylinositol 3-kinase
ROS: Reactive oxygen species
SELE: E-selectin
scRNA-seq: Single-cell RNA-seq
SMCs: Smooth muscle cells
TFs: Transcription factors
THBD: Thrombomodulin
TNF-α: Tumour necrosis factor alpha
