# Supplementary material for: Uncovering emergent phenotypes in endothelial cells by clustering of surrogates of cardiovascular risk factors
Source: Sci Rep. 2022 Jan 25;12:1372. doi: 10.1038/s41598-022-05404-7 (PMC8789842; doi:10.1038/s41598-022-05404-7)
Supplement: Supplementary file 4 — Supplementary Legends. [file 41598_2022_5404_MOESM4_ESM.pdf]

Supplementary Table 1. Differentially expressed genes (DEGs) identified from the comparison of OSS & Hyp & IL1B & OxPAPC vs. LSS (620 DEGs in total)

Supplementary Table 2. scRNA-seq cell type annotation DEGs from human coronary artery plaques

Supplementary Table 3. DEGs identified from the comparison of endothelial cell cluster 2 vs endothelial cell cluster 1 (670 DEGs in total)
