# Supplementary material for: Uncovering emergent phenotypes in endothelial cells by clustering of surrogates of cardiovascular risk factors
Source: Sci Rep. 2022 Jan 25;12:1372. doi: 10.1038/s41598-022-05404-7 (PMC8789842; doi:10.1038/s41598-022-05404-7)
Supplement: Supplementary file 5 — Supplementary Information 5. [file 41598_2022_5404_MOESM5_ESM.pdf]

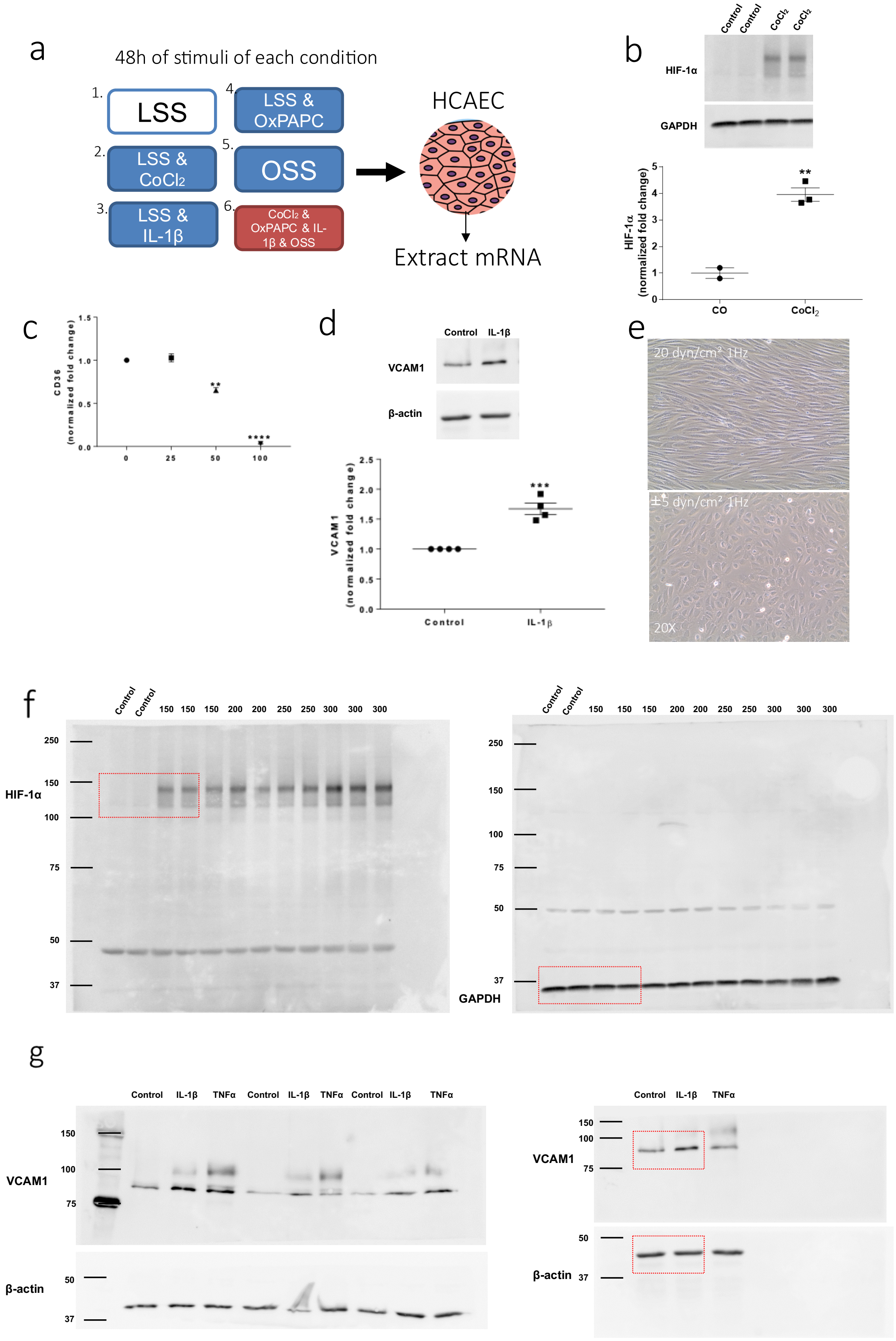
a
48h of stimuli of each condition
1. LSS
2. LSS & CoCl2
3. LSS & IL-1β
4. LSS & OxPAPC
5. OSS
6. CoCl2 & OxPAPC & IL-1β & OSS
HCAEC
Extract mRNA
b
Control
Control
CoCl2
CoCl2
HIF-1α
GAPDH
HIF-1α (normalized fold change)
CO
CoCl2
c
CD36 (normalized fold change)
0
25
50
100
d
Control
IL-1β
VCAM1
β-actin
VCAM1 (normalized fold change)
Control
IL-1β
e
20 dyn/cm² 1Hz
±5 dyn/cm² 1Hz
20X
f
HIF-1α
GAPDH
g
VCAM1
β-actin
Control
IL-1β
TNFα

Supplementary Fig 1. ***In vitro*** **shear stress in human coronary artery endothelial cell (HCAEC) model and further standardization of each stimuli. a** Overall design of HCACE submitted to each stimulus, either individual or combined . **b** HCAEC under static condition stimulated with $CoCl_2$ for 48 h, and then HIF-1α protein expression was determined by Western blot (**p<0.01, *vs.* static control**,** n=3), GAPDH served as a housekeeping protein. **c** HCAEC under static condition simulated by OxPAPC by 48h, RNA collected was reverse transcribed and cDNA was used for real-time PCR quantification of *CD36* and *ATF3* using GAPDH as an internal control (**p<0.01, ***p <0.001, ****p<0.0001 *vs.* static control, n=2). **d** HCAEC under static condition stimulated with IL-1β, and then VCAM1 protein expression was determined by Western blot (***p<0.001 *vs.* static control, n=4), served as a housekeeping protein β-actin. **e** HCAEC under LSS and OSS for 48h showing an aligned cell pattern in LSS when compared to EC under OSS. Data are presented as mean ± SEM; p values were determined by Student's t-test (**c**,**d**) and One way-Anova (**c**). **f** HCAEC under static condition stimulated with CoCl2 (100, 200, 250 or 300μM) for 48 h, and then HIF-1α protein expression was determined by Western blot. GAPDH served as a housekeeping protein. **g** HCAEC under static condition stimulated with IL-1β and TNFα, and then VCAM1 protein expression was determined by Western blot. β-actin served as a housekeeping protein**.** Figure **a** created in the Mind the Graph platform (www.mindthegraph.com).

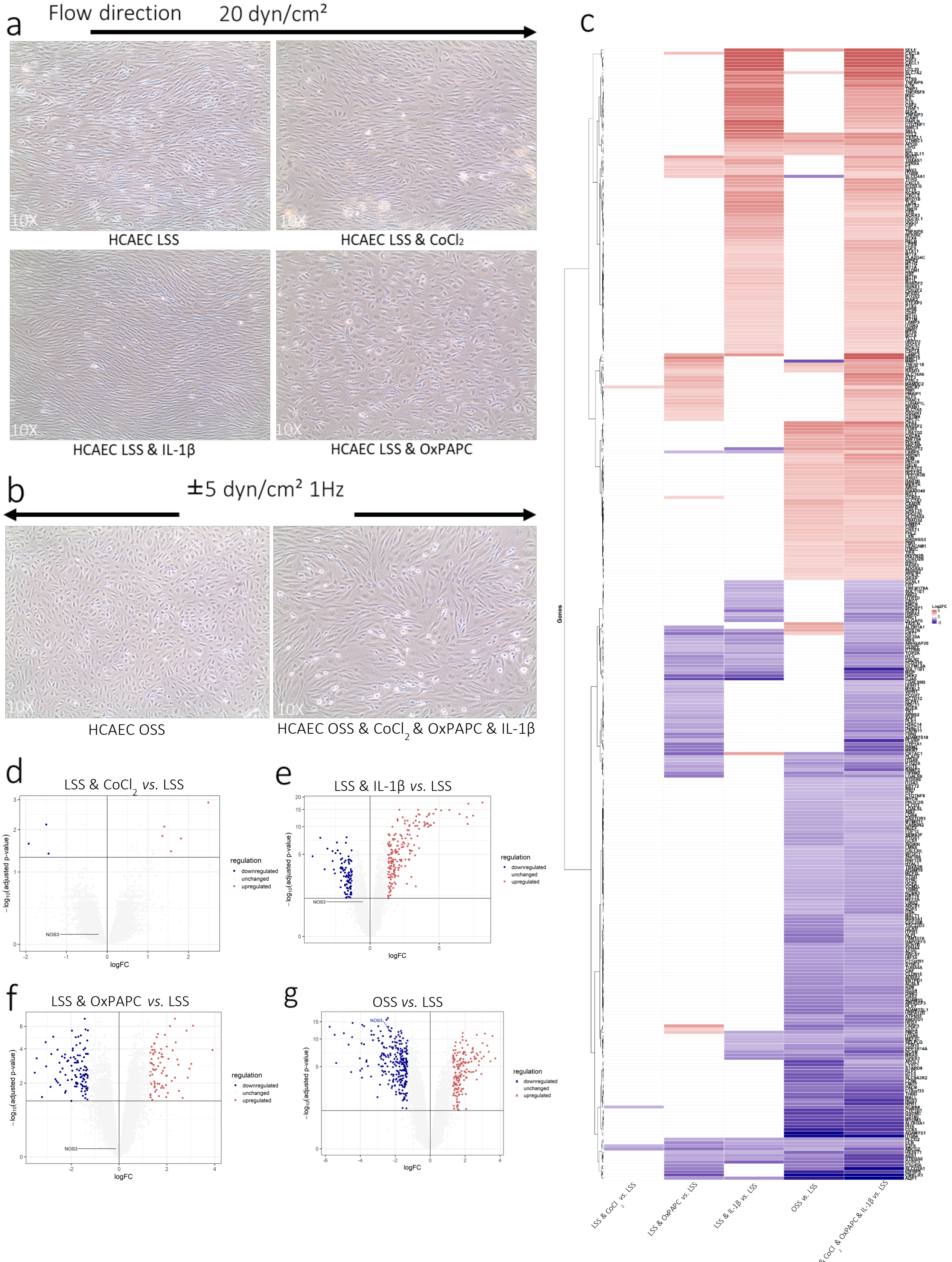

Supplementary Fig 2. Human coronary artery endothelial cell (HCAEC) under the $CoCl_2$, IL-1β, OxPAPC and OSS and their differentially expressed genes (DEGs) when compared to LSS. a HCAEC submitted to unidirectional LSS (20dyn/cm²) alone or combined with $CoCl_2$ (150µM), IL-1β (10ng/ml) and OxPAPC (40µg/ml) by 48h. **b**

HCAEC submitted to bidirectional OSS (± 5dyn/cm² 1Hz) alone or together with $CoCl_2$, IL-1β, and OxPAPC. **c** Heatmap of 349 DEGs of the clustered risk factors that are differentially expressed in the individual condition. Example of stimuli-dependent genes red arrows. **d-g** Volcano plots showing the DEGs when comparing each stimulus to LSS. The DEGs were considered as adjusted *p*-value < 0.05 and $|Log2foldchange| > 1.3$ highlighting the expression of *NOS3*.

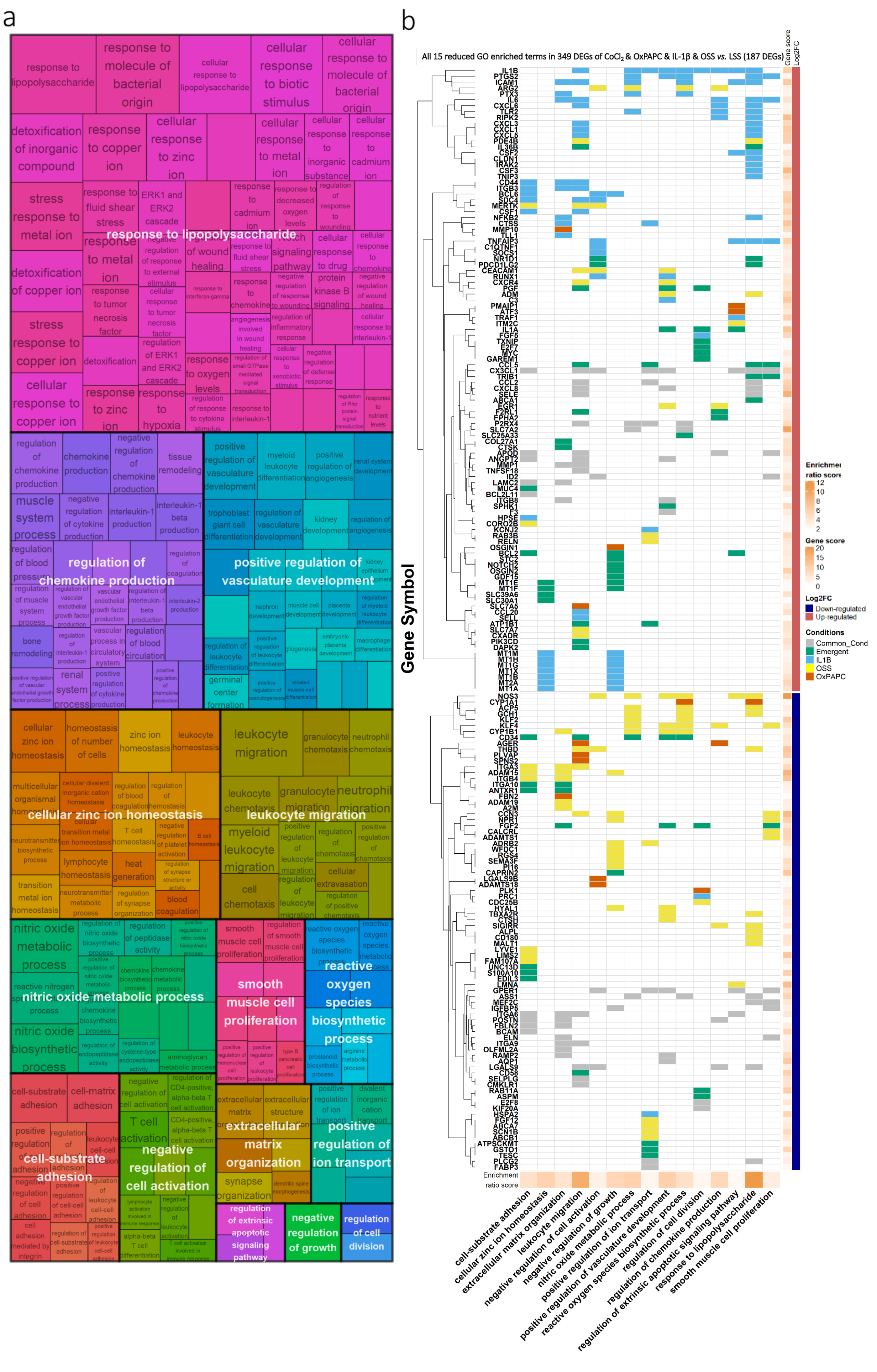
a
b
response to lipopolysaccharide
chemokine production
positive regulation of vasculature development
cellular zinc ion homeostasis
leukocyte migration
nitric oxide metabolic process
smooth muscle cell proliferation
reactive oxygen species biosynthetic process
cell-substrate adhesion
negative regulation of cell activation
extracellular matrix organization
positive regulation of ion transport
regulation of extrinsic apoptotic signaling pathway
negative regulation of growth
regulation of cell division
All 15 reduced GO enriched terms in 349 DEGs of CoCl2 & OxPAPC & IL-1β & OSS vs. LSS (187 DEGs)
Gene score
Log2FC
Gene Symbol
Enrichment ratio score
Enrichment ratio score
12
10
8
6
4
2
Gene score
20
15
10
5
0
Log2FC
Down-regulated
Up-regulated
Conditions
Common_Cond
Emergent
IL1B
OSS
OxPAPC
cell-substrate adhesion
cellular zinc ion homeostasis
extracellular matrix organization
leukocyte migration
negative regulation of cell activation
negative regulation of growth
nitric oxide metabolic process
positive regulation of ion transport
positive regulation of vasculature development
reactive oxygen species biosynthetic process
regulation of cell division
regulation of chemokine production
regulation of extrinsic apoptotic signaling pathway
response to lipopolysaccharide
smooth muscle cell proliferation

Supplementary Fig 3. Gene Ontologies (GO) reduced enriched terms for the 620 differentially expressed genes (DEGs) of the clustered risk factors *vs.* LSS. a GO enrichment analysis followed by redundancy analysis identified and kept the most representative term from the redundant terms (cutoff = 0.9 and adjusted p-value ≤ 0.05). Gene score and enrichment risk score were calculated by $-\log_{10}$(adjusted *p*-value). The DEGs considered were adjusted as *p*-value ≤ 0.05 and |Log2foldchange| ≥ 1.3. **b** Canonical enriched terms in $CoCl_2$ & IL1B & OxPAPC & OSS *vs.* LSS (121 DEGs) and the source of the stimulus individual (represented by colours light blue, yellow and orange), common (grey), more than one stimulus and if it is emergent, meaning that the gene is only altered if all four stimuli are combined (green). Gene score and enrichment risk score were calculated by $-\log_{10}$(adjusted *p*-value).

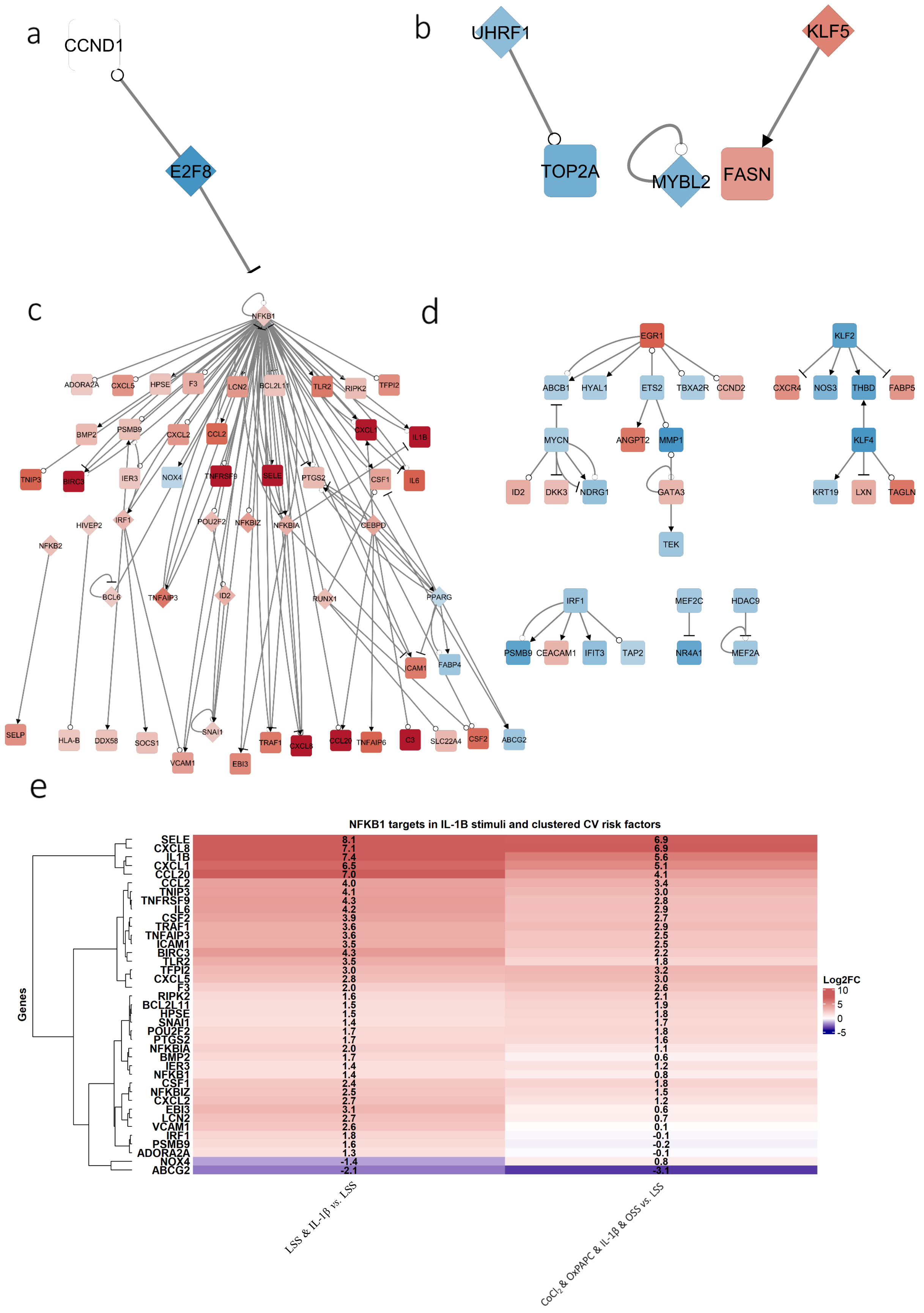
a
CCND1
E2F8
b
UHRF1
TOP2A
MYBL2
FASN
KLF5
c
NFKB1
ADORA2A
CXCL5
HPSE
F3
LCN2
BCL2L11
TLR2
RIPK2
TFPI2
BMP2
PSMB9
CXCL2
CCL2
CXCL1
IL1B
TNIP3
BIRC3
IER3
NOX4
TNFRSF9
SELE
PTGS2
CSF1
IL6
HIVEP2
IRF1
POU2F2
NFKBIZ
NFKBIA
CEBPD
NFKB2
BCL6
TNFAIP3
ID2
RUNX1
PPARG
ICAM1
FABP4
SELP
HLA-B
DDX58
SOCS1
VCAM1
SNAI1
EBI3
TRAF1
CXCL8
CCL20
TNFAIP6
C3
SLC22A4
CSF2
ABCG2
d
EGR1
ABCB1
HYAL1
ETS2
TBXA2R
CCND2
MYCN
ANGPT2
MMP1
ID2
DKK3
NDRG1
GATA3
TEK
KLF2
CXCR4
NOS3
THBD
FABP5
KLF4
KRT19
LXN
TAGLN
IRF1
PSMB9
CEACAM1
IFIT3
TAP2
MEF2C
NR4A1
HDAC9
MEF2A
e
NFKB1 targets in IL-1B stimuli and clustered CV risk factors
Genes
Log2FC
10
5
0
-5
SELE 8.1 6.9
CXCL8 7.1 6.9
IL1B 7.4 5.6
CXCL1 6.5 5.1
CCL20 7.0 4.1
CCL2 4.0 3.4
TNIP3 4.1 3.0
TNFRSF9 4.3 2.8
IL6 4.2 2.9
CSF2 3.9 2.7
TRAF1 3.6 2.9
TNFAIP3 3.6 2.5
ICAM1 3.5 2.5
BIRC3 4.3 2.2
TLR2 3.5 1.8
TFPI2 3.0 3.2
CXCL5 2.8 3.0
F3 2.0 2.6
RIPK2 1.6 2.1
BCL2L11 1.5 1.9
HPSE 1.5 1.8
SNAI1 1.4 1.7
POU2F2 1.7 1.8
PTGS2 1.7 1.6
NFKBIA 2.0 1.1
BMP2 1.7 0.6
IER3 1.4 1.2
NFKB1 1.4 0.8
CSF1 2.4 1.8
NFKBIZ 2.5 1.5
CXCL2 2.7 1.2
EBI3 3.1 0.6
LCN2 2.7 0.7
VCAM1 2.6 0.1
IRF1 1.8 -0.1
PSMB9 1.6 -0.2
ADORA2A 1.3 -0.1
NOX4 -1.4 0.8
ABCG2 -2.1 -3.1
LSS & IL-1β vs. LSS
CoCl2 & OxPAPC & IL-1β & OSS vs. LSS

Supplementary Fig 4. Transcription factors (TFs) regulatory network of each stimulus. **a** $CoCl_2$ & LSS *vs.* LSS regulatory network. **b** OxPAPC & LSS *vs.* LSS regulatory network. **c** IL-1β & LSS *vs.* LSS regulatory network. **d** OSS *vs.* LSS regulatory network. Using the TRRUST database (version 2), the TFs were mapped to their published transcriptional targets. The identified transcriptional network was further filtered based on whether the TF and their targets were a DEG (adjusted *p*-value ≤ 0.05 and $|\text{Log2foldchange}| \geq 1.3$). The diamond shape is TF and the square is the DEG target. The edges mean arrow (activation), circle (unknow) and bar (repression). Node size according to the degree. Genes in blue are downregulated, genes in red upregulated. Green is a modulated emergent gene; Orange is an OxPAPC modulated gene; Light blue is an IL-1β modulated gene; Yellow is an OSS modulated gene; Gray when the gene is modulated in more than one condition. **e** Heatmap of 38 target DEGs from the TF NFKB1 and their log2foldchange in LSS & IL-1β *vs.* LSS and $CoCl_2$ & OxPAPC & IL1B & OSS *vs.* LSS.

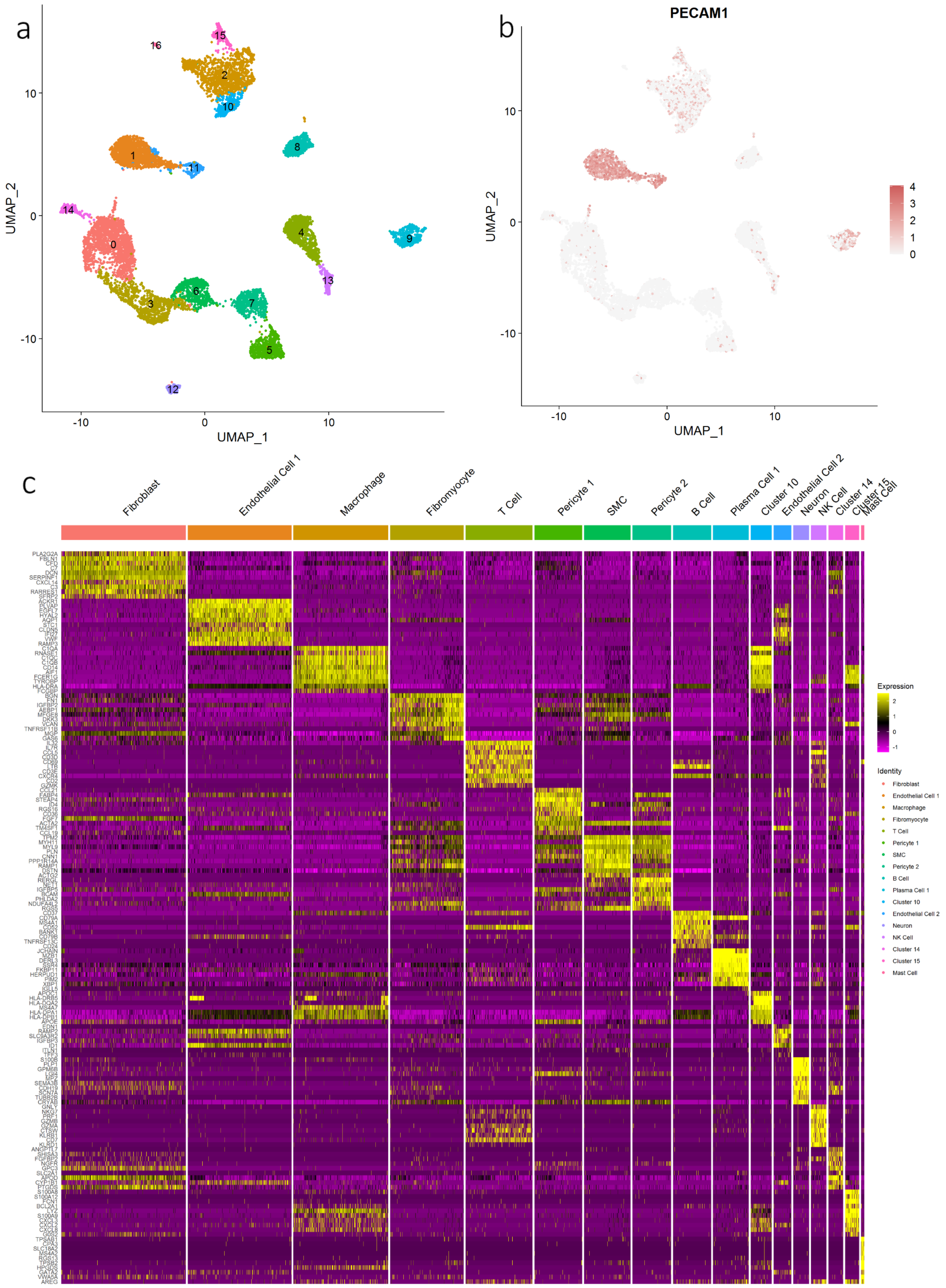

a
UMAP_1
UMAP_2
b
PECAM1
UMAP_1
UMAP_2
c
Fibroblast
Endothelial Cell 1
Macrophage
Fibromyocyte
T Cell
Pericyte 1
SMC
Pericyte 2
B Cell
Plasma Cell 1
Cluster 10
Endothelial Cell 2
Neuron
NK Cell
Cluster 14
Cluster 15
Mast Cell
Expression
Identity

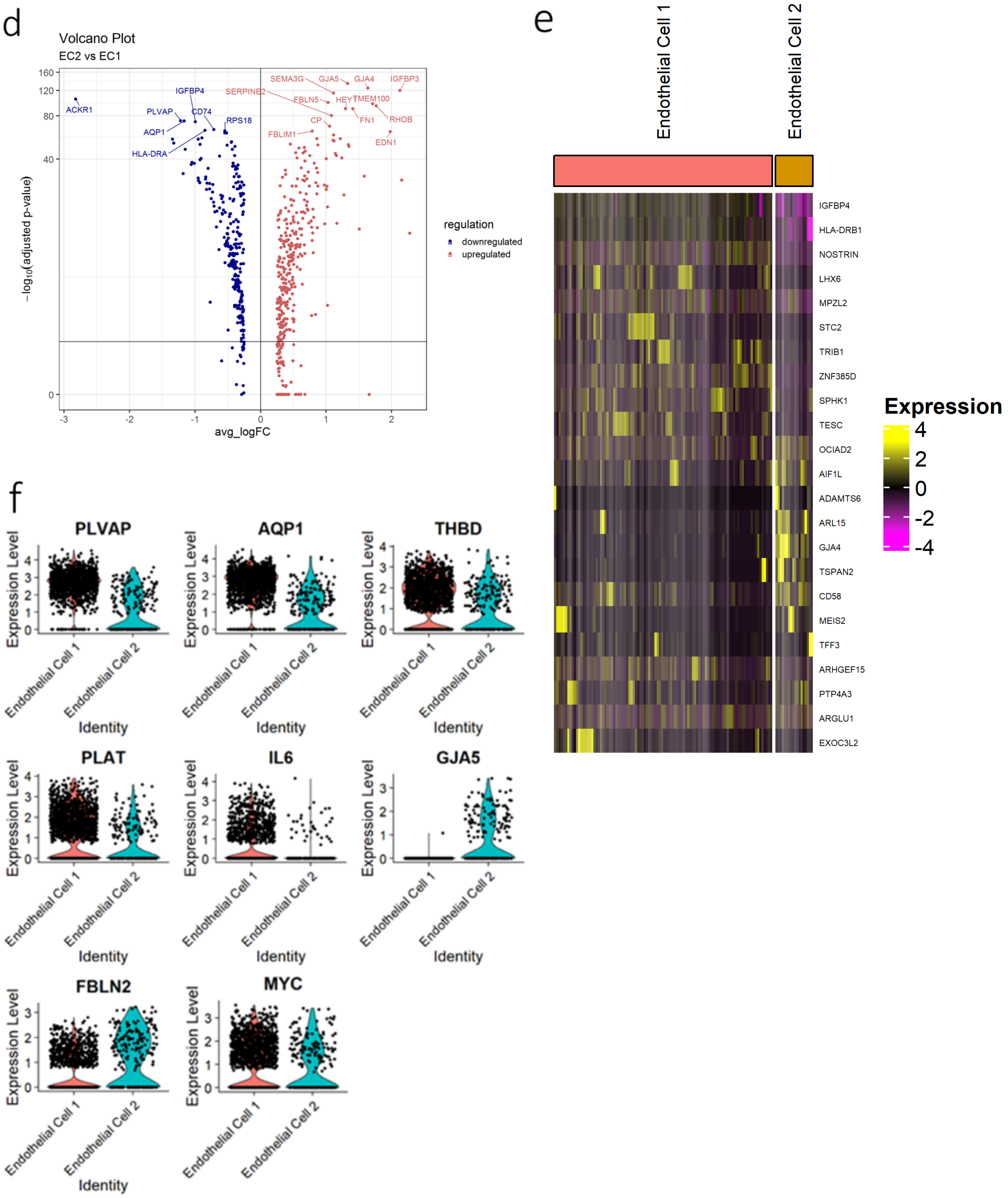

Supplementary Fig 5. a UMAP visualization of clustering identified 17 cell populations (n = 10,671 cells) using the cluster marker genes found with Seurat's function FindAllMarkers(), which finds differentially expressed genes between a cluster and all remaining cells. DEG were considered as adjusted p-value ≤ 0.05 and |Log2foldchange| ≥ 0.25. **b** UMAP visualization of a key endothelial cell (EC) marker *PECAM1* (CD31). **c** Heatmap of the top 10 gene markers expression levels from each cell in the 17 cell clusters. **d** Volcano plots showing the differentially expressed genes (DEGs) when comparing EC2 *vs.* EC1. The DEGs were considered as adjusted p-value < 0.05 and |Log2foldchange| > 0.25 highlighting the top 20 DEGs. **e** Heatmap of the emergent DEGs expression levels in which they are differentially expressed in the comparison EC2 *vs.*

EC1. **f** Violin plot of canonical clustered risk factors DEGs which are differentially expressed in EC2 *vs*. EC1.
